# An investigation of the relationship between alarm and compassion fatigue in surgical intensive care nurses: a cross-sectional study

**DOI:** 10.1186/s12912-025-03828-w

**Published:** 2025-09-30

**Authors:** Hamdiye Banu Katran, Barış Özdere, Fatma Eti Aslan

**Affiliations:** 1 Marmara University Faculty of Health Sciences, İstanbul, Turkey; 2 Mus Alparslan University Health Services Vocational School, Muş, Turkey; 3 Bahcesehir University Graduate School of Education, Istanbul, Turkey; 4 Bahçeşehir University Faculty of Health Sciences, İstanbul, Turkey

**Keywords:** Alarm fatigue, Compassion fatigue, Critical care, Intensive care, Nursing

## Abstract

**Background:**

Particularly, the medical devices utilized in specialized units such as intensive care units, along with their associated alarm sounds, can potentially induce compassion fatigue among nursing professionals. This study was planned to investigate the relationship between alarm and compassion fatigue in nurses working in surgical intensive care units.

**Methods:**

This descriptive, cross-sectional correlational study (July–August 2024) surveyed 162 surgical ICU nurses from a university and a state hospital in eastern Türkiye. Using a proportionally stratified, consecutive (non-probability) sampling strategy, the team approached every on-duty nurse meeting inclusion criteria (≥ 1 year SICU experience), achieving an 85.7% response rate (*N* = 162; G*Power-derived minimum *n* = 151). Researchers collected data via face-to-face interviews using a 14-item Demographic Form, the 10-item Alarm Fatigue Scale (α = 0.77), and the 13-item Compassion Fatigue Short Scale (α = 0.91). After ethical approval and informed consent, responses were analysed in SPSS 27.0: normality (Kolmogorov-Smirnov, skewness/kurtosis), descriptive statistics, Pearson correlations (*with Cohen’s effect sizes), and multivariate linear regression (adjusting for (covariates)), with reliability assessed via Cronbach’s α; significance was set at *p* < 0.05. Missing data (< 5%) were excluded listwise.

**Results:**

The mean alarm fatigue score of surgical intensive care nurses was 23.77 ± 7.26 and the mean compassion fatigue score was 62.82 ± 26.66. A moderate positive significant relationship was found between alarm fatigue and compassion fatigue (*r* = 0.302, *p* < 0.01). Regression analysis showed that alarm fatigue predicted compassion fatigue by 9% (R²=0.091, *p* < 0.05). No significant correlation was found between sociodemographic factors and alarm and compassion fatigue levels.

**Conclusions:**

Alarm fatigue was found to be an important determinant of compassion fatigue. Nurses were found to have moderate levels of alarm and compassion fatigue. This may negatively affect nurses’ quality of patient care and job satisfaction. It is recommended that healthcare organizations increase alarm management training and prioritize strategies that support nurses’ well-being. These steps can improve patient care outcomes by reducing nurses workload.

## Introduction

Intensive care units (ICUs) represent the most technologically advanced and critical environments within healthcare institutions where patients receive continuous and complex care. Nurses working in these units play a pivotal role in improving patient outcomes. When high-quality care is delivered, it not only contributes to patients physical, emotional, and psychological recovery, but also enhances nurses’ sense of professional fulfillment. This positive feeling, referred to as compassion satisfaction, is defined as pleasure derived from being able to do one’s work well, particularly in helping others in distress. However, ICU nurses are frequently exposed to high patient acuity, increased emotional demands from patients and their families, and continuous exposure to life-threatening situations. These factors, coupled with heavy workloads and suboptimal working conditions, can lead to compassion fatigue. First described in the 1990s among emergency nurses, compassion fatigue is a psychosocial phenomenon that results in emotional exhaustion, reduced empathy, and a diminished capacity for compassion due to prolonged and repeated exposure to patient suffering and trauma [1–4].

In recent years, healthcare systems have increasingly adopted advanced technologies to improve care quality and ensure patient safety [5]. Among these technologies are a wide range of medical devices equipped with visual and auditory alarms, especially in critical care environments, such as surgical ICUs [6, 7]. Devices such as monitors, ventilators, infusion pumps, and oxygen saturation systems continuously emit alarms to alert clinicians to deviations in vital signs or technical malfunction. These alarms are essential for timely intervention; however, when multiple devices generate frequent and often false or nonactionable alarms, nurses may become desensitized or overwhelmed, a condition termed *alarm fatigue* [8, 9]. Alarm fatigue is characterized by diminished responsiveness to clinically significant alarms owing to excessive exposure to alarm sounds. The contributing factors include high false alarm rates, persistent noise, human factors, and organizational shortcomings [8, 10, 11]. Although clinical alarms are designed to enhance patient safety and are integral to international healthcare quality standards, poor alarm management can paradoxically pose a threat to patient safety [12]. In response to constant auditory stimuli, nurses may engage in unsafe practices such as silencing or disabling alarms, lowering alarm thresholds, or ignoring alerts altogether. In particular, surgical ICU nurses may experience heightened stress and cognitive overload due to continuous exposure to alarm systems, ultimately affecting their performance and quality of care delivered [13].

Given that surgical ICU nurses are expected to act swiftly and accurately in response to clinical alarms, understanding how alarm fatigue contributes to or exacerbates compassion fatigue is essential. The combination of emotional exhaustion and sensory overload can have serious consequences for patient safety and nurse well-being [9]. Given the critical importance of this subject, there is a notable paucity of comprehensive studies in the current literature that thoroughly investigate the potential relationship between compassion fatigue and alarm fatigue among intensive care nurses, particularly those employed in surgical care units. A comprehensive review from 2025 still characterizes alarm fatigue as “a phenomenon that has not been sufficiently researched” [14], and studies that quantitatively examine both types of fatigue concurrently are exceedingly rare, with one of the most prominent being the research conducted by Storm and Chen involving mixed intensive care and step-down unit nurses [9] A limited number of studies in the literature report that the daily number of alarms per bed ranges from 119 to 350 in different sample groups, indicating that nurses are consistently subjected to an intense barrage of auditory stimuli [15–18]. This relentless sensory load, particularly in an ethically sensitive and highly critical postoperative environment, facilitates both sensory overload and empathic burnout.

By investigating the relationship between alarm fatigue and compassion fatigue among nurses working in the SICUs of two hospitals in eastern Turkey, this study aimed to address a significant contextual and geographical gap. The findings obtained are anticipated to contribute directly to the development of intervention programs aimed at enhancing the professional well-being of nurses providing frontline postoperative care and improving patient safety.

## Methods

### Purpose and type of study

This descriptive, cross-sectional, and correlational study was conducted to investigate the relationship between alarm fatigue and compassion fatigue among nurses employed in intensive care units (ICUs) where surgical patients were monitored.

### Population and sample of the study

The target population comprised all nurses (*N* = 189) working in the surgical intensive care units (SICUs) of one university hospital (*n* = 105) and one state hospital (*n* = 84) located in two cities in eastern Türkiye. The minimum sample size was calculated with G*Power using the finite-population correction (5% margin of error, 95% confidence level, medium effect size), which indicated that 151 nurses—82 from the university hospital and 69 from the state hospital—would be sufficient.

A non-probability, proportionally stratified consecutive sampling approach was adopted. Between July and August 2024, the research team made repeated site visits on different days to approach all eligible nurses who were on duty, thereby minimising disruption to unit workflows and maximising coverage of the accessible population. Ultimately, usable data were obtained from 162 SICU nurses (response rate = 85.7%), of whom 90 worked at the university hospital and 72 at the state hospital. Collecting slightly more participants than the calculated minimum helped increase statistical power and reduce the impact of incomplete or inaccurately completed questionnaires.

#### Inclusion criteria

- Registered nurses currently employed in a surgical intensive care unit (SICU).
- At least 12 months of cumulative SICU experience prior to data collection.

#### Exclusion criterion

- Registered nurses with < 12 months of total SICU experience.

### Data collection tools and method

The “Descriptive Information Form,” “Alarm Fatigue Scale,” and “Compassion Fatigue Short Scale (CFSS)” were utilized for data collection. Following the acquisition of requisite approval from the ethics committee and pertinent institutions, data were gathered from surgical intensive care nurses who were informed of the study and provided written informed consent. The data collection process was conducted between July 2024 and August 2024, with nurses employed in the surgical intensive care units (anesthesia, general, emergency, cardiovascular surgery, neurosurgery, and general surgery intensive care) of two distinct hospitals situated in eastern Turkey. To mitigate data loss, researchers considered nurses’shift patterns and devised a plan to avoid disrupting operations. Data were obtained through individual face-to-face interviews, each lasting approximately 5–10 min.

### Introductory information form

The introductory information form includes information regarding the literature on the subject [19–24] prepared by the researchers based on. The questionnaire comprises 14 items, including demographic information such as age, gender, marital status, educational attainment, occupation, specific intensive care unit of employment, years of experience in intensive care, years of professional experience, shift duration, average monthly work hours, number of patients under care, professional satisfaction, level of distress caused by alarms, and participation in alarm systems and management training. The instrument incorporates one item evaluated using a numerical rating scale (0–2). The item assessing nurses’ level of distress due to alarms was measured on a 3-point Likert scale, with “0” indicating “No distress,” “1” indicating “Partial distress,” and “2” indicating “Severe distress.”

### Alarm fatigue scale (AFS)

The scale developed by Torabizadeh et al. (2017) was used to assess alarm-induced psychological pressure in nurses employed in intensive care units. Alan et al. (2021) conducted a Turkish validity and reliability study [19, 25]. The scale employs a 5-point Likert scale, ranging from “0” (never) to “4” (always). The potential scores on the scale ranged from 0 to 48, with higher scores indicating greater alarm fatigue experienced by nurses. The scale comprises two sub-dimensions: a positive response sub-dimension that encompasses clinical practices to reduce alarms, and a negative response sub-dimension that includes practices to increase alarms. The scale contains no reverse items. Cronbach’s alpha coefficient were 0.71 for the total scale, 0.63 for the positive reaction sub-dimension, and 0.74 for the negative reaction sub-dimension, respectively [19, 25]. In this study, the Cronbach’s alpha internal consistency coefficient of the Alarm Fatigue Scale was 0.77.

### Compassion fatigue short scale (CF-SS)

The scale was developed by Adams et al. (2006) [26] and subsequently adapted to Turkish by Dinç and Ekinci (2019) [27]. This self-report assessment instrument requires participants to indicate the degree to which each item reflects their experience. The scale employs a 10-point Likert-type format, ranging from rarely/never (1) to very often (10). Comprising 13 items, the scale consists of two sub-dimensions: secondary trauma and occupational burnout. Items “c, e, h, j, l” in the scale measure secondary trauma, while items “a, b, d, f, g, i, k, m” assess occupational burnout. The Cronbach’s alpha coefficients of the sub-dimensions in the original scale ranged from 0.80 to 0.90, and those of the adapted scale ranged from 0.88 to 0.85. The minimum obtainable score was 13 and the maximum score was 130. Higher scores indicate a greater level of compassion fatigue experienced by individuals [26, 27]. In this study, Cronbach’s alpha for the internal consistency coefficient of the Compassion Fatigue Scale was 0.91.

### Statistical analysis

The Statistical Package for the Social Sciences (SPSS 27) statistical analysis program was used to evaluate the data. This study employed descriptive analyses including frequency, percentage, mean, and standard deviation. The Kolmogorov-Smirnov test was applied to determine whether the variables were normally distributed, and the results showed that the data were normally distributed (*p* > 0.05). If the sample size exceeds 50 and the Skewness and Kurtosis values fall within the range of -1.5 to + 1.5, the data can be considered to follow a normal distribution [28]. The Alarm Fatigue Scale (skewness: 0.230, kurtosis: 0.285) and Compassion Fatigue Scale (skewness: 0.371, kurtosis: − 0.554) were determined to exhibit a normal distribution. Correlation coefficients (r) were interpreted as high (≥ 0.60), moderate (0.30 – 0.59), or weak (< 0.30),; and 0, no correlation [29]. Pearson’s correlation analysis was employed to ascertain the relationship between variables, while linear regression analysis was used to assess the effect of the independent variable on the dependent variable. The Cronbach’s alpha was used to evaluate the reliability of the scale.

## Results

Upon examination of the distribution of descriptive characteristics among the nurses included in the study, 56.8% of the participants were between 27 and 34 years of age, 61.7% were male, 53.1% were married, and 61.1% held a Bachelor’s degree. Furthermore, 53.7% had been employed in the intensive care unit for 1–4 years, while 48.1% had 5–8 years of professional experience. The majority (93.2%) worked both day and night shifts, with 84% working 24-hour shifts. Additionally, 49.4% worked between 160 and 208 h per month, and 70.4% were responsible for to 1–2 patients. Regarding job satisfaction, 51.9% of participants reported that they enjoyed their work. Regarding alarm-related issues, 49.4% were partially disturbed by alarms and 64.8% had not received training on alarm systems (Table 1).

**Table 1:** Descriptive characteristics of nurses (*N* = 162)

Variables	Number	%
**Age**		
22–26	42	25.9
27–34 35 and above	92 28	56.8 17.3
**Sex**		
Female	62	38.3
Male	100	61.7
**Marital status**		
Married	86	53.1
Single	76	46.9
**Education level**		
Secondary Education Associate degree License Undergraduate	27 27 99 9	16.7 16.7 61.1 5.5
**Intensive care working time**		
1–4 year 5–8 year 9 years and above	87 55 20	53.7 34.0 12.3
**Length of service in the profession**		
1–4 year 5–8 year 9 years and above	40 78 44	24.7 48.1 27.2
**Mode of operation**		
Day/night Daytime only	151 11	93.2 6.8
**Shift pattern**		
8- hour shift 16- hour shift 24- hour shift	17 9 136	10.5 5.5 84.0
**Monthly working time**		
160–208 clock 209–256 clock 257–304 clock	80 59 23	49.4 36.4 14.2
**Number of patients for whom he/she is responsible**		
1–2 3–4	114 48	70.4 29.6
**Doing your job with love**		
Yes No	84 78	51.9 48.1
**Alarm-related discomfort**		
I’m not bothered at all I am partially disturbed I feel very uncomfortable	19 80 63	11.7 49.4 38.9
**Status of receiving training on alarm systems**		
Yes No	57 105	35.2 64.8

No statistically significant correlation was observed between the mean scores on the alarm and compassion fatigue scales of the participants and the variables of age, sex, educational status, marital status, shift type, years of experience in intensive care, years of professional experience, average monthly working hours, and number of patients under care.

Analysis of the mean scores for the alarm fatigue and compassion fatigue scales revealed that the mean scores of the alarm fatigue sub-dimensions were 11.20 ± 2.73 for positive reaction, 12.56 ± 6.28 for negative reaction, and 23.77 ± 7.26 for total alarm fatigue. The mean scores of compassion fatigue sub-dimensions were 22.36 ± 10.93 for secondary trauma, 40.46 ± 17.52 for occupational burnout, and 62.82 ± 26.66 for total compassion fatigue (Table 2).

**Table 2:** Distribution of mean total scores obtained by nurses from the alarm fatigue and compassion fatigue scales

Scale		Lowest	Highest	Score average
Alarm fatigue	Positive Response	0.00	16.00	11.20 ± 2.73
	Negative Reaction	0.00	29.00	12.56 ± 6.28
	**Total**	0.00	45.00	23.77 ± 7.26
Compassion fatigue	Secondary Trauma	6.00	50.00	22.36 ± 10.93
	Occupational Burnout	8.00	80.00	40.46 ± 17.52
	**Total**	14.00	130.00	62.82 ± 26.66

Examination of the relationship between nurses’ alarm fatigue and compassion fatigue mean scores revealed a weak negative correlation between positive reactions and occupational burnout (*r*=-0.204, *p* < 0.05) and compassion fatigue total mean scores (*r*= -0.158, *p* < 0.05), while no significant correlation was found with secondary trauma (*r*= -0.059, *p* > 0.05). A moderately significant positive correlation was identified between negative reactions and secondary trauma (*r* = 0.419, *p* < 0.01), occupational burnout (*r* = 0.374, *p* < 0.01), and compassion fatigue total mean score (*r* = 0.417, *p* < 0.01). Furthermore, a moderate positive significant correlation was observed between the mean total score of alarm fatigue and the mean total score of compassion fatigue (*r* = 0.302, *p* < 0.01) (Table 3).

**Table 3:** The relationship betweennurses’ alarm fatigue and compassion fatigue levels

		Secondary trauma	Compassion fatigue	
			Occupational burnout	Total
Alarm fatigue	Positive	*r* = − 0.059	*r* = − 0.204*	*r* = − 0.158*
	Response	*p* = 0.455	*p* = 0.009	*p* = 0.044
	Negative	*r* = 0.419**	*r* = 0.374**	*r* = 0.417**
	Reaction	*p* = 0.000	*p* = 0.000	*p* = 0.000
	Total	*r* = 0.340**	*r* = 0.247*	*r* = 0.302**
		*p* = 0.000	*p* = 0.002	*p* = 0.000

*p < 0.05 and **p < 0.001 significant

The regression analysis results indicated that nurses alarm fatigue levels had a positive and weakly significant effect on their compassion fatigue. The explanatory power of the model, expressed as R^2^ value, was 0.091 (F = 16.04; R^2^ = 0.091; *p* < 0.05). This value indicates that 9% of the variance in the compassion fatigue variable is explained by the independent variable in the model, namely alarm fatigue. The Beta coefficient of the independent variable included in the regression model was = 0.30 (*p* < 0.05). Consequently, alarm fatigue had a significant effect on compassion fatigue levels (*p* < 0.05) (Table 4).

**Table 4:** Regression model of alarm fatigue predicting compassion fatigue

	B	Standard error	β	t	*p*	Durbin–Watson	VIF
(Constant)	36.476	6.877		5.304	< 0.001*	1.686	
Alarm fatigue	1.109	0.277	0.302	4.005	< 0.001*		1.00

R^2^ = 0.091; Adj. R^2^ = 0.085; F = 16.043; *p* < 0.001*

Note: Dependent variable, Compassion Fatigue Scale. Abbreviations: Adj. R^2^, adjusted coefficient of determination; B, non-standardized beta value; Durbin–Watson, autocorrelation coefficient; F, significance of variable; R^2^, coefficient of determination; t, significance of variable; VIF, variance inflation factor; β, standardized beta value. *p < 0.001

## Discussion

This section discusses the findings of the analysis and compares them with those of previous studies on alarm and compassion fatigue. No other study conducted in Turkey has utilized the two scales employed in this study. Consequently, comparisons were made with similar studies.

This study revealed no statistically significant correlation between nurses’sociodemographic characteristics and their levels of alarm and compassion fatigue. A study conducted in Turkey demonstrated that the mean score of the alarm fatigue scale was not significantly influenced by nurses’ length of service in the profession and intensive care unit nor by shift-type variables [30]. In a study conducted with hemodialysis nurses, Sun et al. found that demographic characteristics such as years of experience, number of patients managed per shift, and whether specialized nursing training was received demonstrated statistically significant relationships with alarm fatigue (*p* < 0.05) [31]. Storm and Chen’s study revealed a significant relationship between alarm fatigue in intensive care nurses and several factors, including sex, care unit, nurse-patient ratio, and age characteristics [9].

Alarm fatigue is a phenomenon characterized by delayed or absent responses to clinical monitor alarms due to desensitization of nurses resulting from excessive monitoring alarms. Intensive care units are the primary hospital units that contribute to alarm fatigue owing to their high-intensity, stressful patient care environments and diverse clinical monitor alarms [6, 32]. In this study, upon examination of the mean scores of the alarm fatigue scale, the mean score was 23.77 ± 7.26, indicating that the alarm fatigue levels of the nurses were below the median level. In various studies utilizing the same scale, it was observed that the mean total score of the alarm fatigue scale ranged from 15.17 ± 5.74 30.1 ± 7.47 [33–37]. Although these results are similar to those of our study, there are also studies in the literature that show higher levels of alert fatigue among nurses. For example, Alkubati et al. (2024) reported that the mean alarm fatigue score was higher among nurses working in critical care settings in Ha’ il, Saudi Arabia. The total mean alarm fatigue score was 26.38 ± 8.30 44, which is above the median level [38]. Similarly, Seok et al. (2023) found a higher mean alarm fatigue score of 28.59 out of 44 ICU nurses in Seoul, Republic of Korea. This score is well above the median and indicates a high degree of alarm fatigue [39]. Differences in the levels of alarm fatigue across studies may be attributed to variations in healthcare systems, work environments, and cultural factors in different countries. Previous research focusing on alarm fatigue among nurses has identified several demographic, professional, and organizational determinants that differ from the findings of the present study. Specifically, gender has been highlighted as a significant factor, with male nurses exhibiting higher levels of alarm fatigue [38, 40]. Likewise, professional experience has been shown to influence outcomes; nurses with fewer years of experience tend to report greater levels of alarm fatigue [38, 41]. Regarding work setting, higher alarm fatigue has been observed among nurses working in neonatal and postoperative intensive care units [34, 38, 41]. In addition, work schedule appears to be relevant, as nurses on 12-hour shifts demonstrate increased levels of alarm fatigue [34, 38]. Psychological factors also play an important role; elevated stress and anxiety levels are consistently associated with greater alarm fatigue. At the organizational level, the presence of a formal alarm management policy has been reported to mitigate alarm fatigue, with lower levels observed in institutions where such policies are implemented [41]. Finally, device- and alarm-related factors contribute substantially to the phenomenon, as increased complexity of alarm parameters and a higher frequency of unnecessary alarms have been shown to exacerbate alarm fatigue [40, 42].

Nurses in intensive care units are responsible for providing continuous care to patients in stressful, critical, and serious health situations. This population is more likely to experience compassion fatigue [43]. The mean total score on the compassion fatigue scale was 62.82 ± 26.66. These scores indicated a moderate level of compassion fatigue among the nurses participating in the study. A review of the literature revealed that intensive care nurses experience moderate to high levels of compassion fatigue, and similar results were obtained in this investigation [2, 9, 44]. This situation can be attributed to the nature of surgical care units and the critical condition of the patients under care in these specialized facilities. The findings of these studies are consistent with those of the present study.

In this study, a moderate positive significant correlation was observed between the mean total scores for alarm fatigue and compassion fatigue. The literature review revealed a paucity of studies that employed both alarm and compassion scales concurrently. Consequently, the discussion in this section is limited. Storm and Chen’s study of 52 intensive care nurses demonstrated that 40% of them experienced alarm fatigue and were at risk of compassion fatigue and burnout [9]. These findings are comparable to those of the present study.

The data indicated that alarm fatigue levels had a positive and weakly significant effect on compassion fatigue, as evidenced by the regression results (R²=0.091; F = 16.04; *p* < 0.05). This finding suggests that approximately 9% of the variance in compassion fatigue can be attributed to alarm fatigue and its role as a contributing factor. The positive correlation between alarm fatigue and compassion fatigue may elucidate the critical challenges nurses encounter in high-stress environments, particularly in intensive care units. Alarm fatigue, characterized by desensitization to frequent alarms from medical devices, can result in decreased attention and responsiveness to patient needs. This phenomenon is of particular concern given the crucial role that nurses play in delivering compassionate care. A beta coefficient of 0.302 for alarm fatigue indicated a significant effect on the levels of compassion fatigue (*p* < 0.05), supporting the assertion that as alarm fatigue increases, so does compassion fatigue among nurses.

Research has consistently demonstrated that alarm fatigue can compromise not only the quality of patient care but also the psychological well-being of healthcare providers. For instance, Storm and Chen emphasized that alarm fatigue contributes to burnout and adversely affects job satisfaction among nurses, which aligns with existing findings on compassion fatigue [9]. Furthermore, Salimi et al. (2020) corroborated these findings by demonstrating that alarm fatigue significantly predicted compassion satisfaction and secondary traumatic stress among intensive care nurses, thereby influencing their overall professional quality of life [45]. Although this study and studies with similar findings suggest a positive relationship between alarm fatigue and compassion fatigue, some studies have not yielded such results. For example, in a study conducted in Korea, no significant relationship was found between alarm fatigue and mental workload in intensive care nurse [39].

In line with Storm and Chen (2021), our findings indicate that alarm fatigue, particularly its negative reaction subdimension, is significantly associated with both secondary trauma and occupational burnout [9]. The moderate correlation coefficients suggest that the cumulative effect of desensitization to auditory stimuli and the emotional burden of care may together erode the psychological resilience of surgical ICU nurses. Despite the modest R² value, the significant association between alarm and compassion fatigue points to a critical relationship worthy of further exploration. It is plausible that other unmeasured variables such as institutional support, coping strategies, or individual resilience could mediate or moderate this relationship, highlighting avenues for future longitudinal research. In conclusion, the relationship between alarm and compassion fatigue appears to be complex, and inconsistent results have been obtained in different studies. This suggests that the relationship between the two phenomena may be influenced by contextual factors and further research is needed. Future studies should focus on examining the potential mediating and moderating factors that influence this relationship.

Given these insights, it is imperative that healthcare organizations implement strategies to reduce alarm fatigue. This may include optimizing alarm settings on medical devices, providing training on effective responses to alarms, and fostering an environment that promotes mental health support for nursing staff. By addressing these factors, healthcare organizations can improve nurses satisfaction and patient care outcomes.

### Limitations

This study has several limitations. First, the sample size was small and confined to hospitals in one geographic region, limiting generalisability. Second, all variables were measured with self-report scales, introducing potential social-desirability and recall bias as well as common-method variance. Third, we did not collect objective alarm counts or physiological fatigue markers, so the findings rely entirely on subjective data. Finally, the cross-sectional design prevents causal inferences; longitudinal studies with multi-site sampling and objective measures are recommended.

## Conclusion

This study investigated the association between alarm and compassion fatigue among nurses employed in surgical care units. The results indicate that the risk of compassion fatigue escalates concomitantly with an increase in alarm fatigue, which adversely affects both nurse performance and the quality of patient care. Consequently, it is imperative for healthcare organizations to prioritize strategies aimed at mitigating alarm fatigue. These strategies encompass the optimization of alarms and the provision of training for nurses on effective alarm management.

Regarding future practices, systematic training programs should be implemented to enable nurses to mitigate unnecessary alerts and respond more efficiently to critical situations. Moreover, fostering an organizational culture that prioritizes mental health and well-being can contribute significantly to alleviating compassion fatigue. By addressing these factors, healthcare organizations can enhance nurses’ job satisfaction and patient outcomes, thereby ensuring a more effective and high-quality healthcare delivery.

### Relevance for clinical practice

In intensive care units (ICUs), patients’ vital signs are continuously monitored via multi-parameter monitors, ventilators, ECMO consoles and dialysis machines. These devices, especially in open-bay ICUs, frequently emit alarms that exceed 70 dB, thereby increasing nurses’ noise exposure. Prolonged exposure fosters alarm fatigue, which our study shows is significantly associated with a higher risk of compassion fatigue. To break this cycle and improve both nurse well-being and patient outcomes, we recommend five evidence-based, unit-level actions:

1. Patient-specific, tiered alarm-priority protocol with limits reviewed at every shift hand-over (critical ≤ 60 s response, advisory ≤ 3 min).
2. Weekly one-click alarm-report audit to identify the top 10% alarm-generating devices for recalibration or service.
3. Low-cost noise-dampening measures (bedside acoustic panels, soft-close lids, rubber-wheeled carts).
4. Bi-annual, two-hour structured alarm-management training and high-fidelity simulation for all nurses, with ≥ 90% competency pass.
5. Well-being programme featuring micro-breaks (≥ 5 min every 2 h in a designated quiet zone) and monthly psychologist-facilitated debrief sessions.

These interventions are feasible within existing quality-improvement cycles and remain effective even in open-bay ICUs.

## Data Availability

Data is provided within the manuscript or supplementary information files.

## Abbreviations

AFS: Alarm Fatigue Scale
CF-SS: Compassion Fatigue Short Scale
ECMO: Extracorporeal Membrane Oxygenation
